# Translating tolerogenic therapies to the clinic – where do we stand?

**DOI:** 10.3389/fimmu.2012.00254

**Published:** 2012-08-20

**Authors:** Fadi Issa, Kathryn J. Wood

**Affiliations:** Transplantation Research Immunology Group, Nuffield Department of Surgical Sciences, Level 6, John Radcliffe Hospital, University of OxfordOxford, UK

**Keywords:** tolerance, immune regulation, cellular therapy, chimerism, regulatory T cell, clinical trials, transplantation

## Abstract

Manipulation of the immune system to prevent the development of a specific immune response is an ideal strategy to improve outcomes after transplantation. A number of experimental techniques exploiting central and peripheral tolerance mechanisms have demonstrated success, leading to the first early phase clinical trials for tolerance induction. The first major strategy centers on the facilitation of donor-cell mixed chimerism in the transplant recipient with the use of bone marrow or hematopoietic stem cell transplantation. The second strategy, utilizing peripheral regulatory mechanisms, focuses on cellular therapy with regulatory T cells. This review examines the key studies and novel research directions in the field of immunological tolerance.

## INTRODUCTION

Strategies to prevent the development of a specific immune response are invaluable in the quest to achieve improved outcomes after solid organ transplantation (SOT), bone marrow and hematopoietic stem cell transplantation (BMT; HSCT), as well as for the treatment of autoimmune diseases. Specific immune unresponsiveness is the hallmark of clinical tolerance, which in turn may be defined as the long-term survival of an allograft with normal function and no evidence for rejection, in the absence of immunosuppressive drug therapy. The quest for tolerance began with the landmark paper by Billingham et al. (1953), in which tolerance was induced to a mouse skin allograft by injection of a recipient mouse with donor-derived F1 cells as a neonate. Current experimental and early clinical strategies to promote tolerance center on the induction of central tolerance by deletion of donor-reactive leukocytes, most commonly the induction of chimerism, or on peripheral tolerance, most commonly the induction or expansion of regulatory T cells (Treg; Wood et al., 2012).

## CHIMERISM

During T cell development in the thymus, T cells with T cell receptors (TCRs) that are strongly reactive to host MHC molecules are deleted by a process termed negative selection (i.e., central deletion). This physiological process has been harnessed experimentally for the induction of tolerance to foreign antigens. The method used by Medawar to achieve tolerance to skin allografts over 60 years ago was in a fortunate strain combination with only a class I MHC mismatch (Billingham et al., 1953). More recently, similar methods have been used to achieve “central deletion” in fully MHC-mismatched models of transplantation (Cober et al., 1999; Butler et al., 2000; Petit et al., 2004; Mathes et al., 2005). Nevertheless, such strategies are neither consistently successful nor easily translatable to the clinic. Alternatively, hematopoietic complete chimerism through myeloablative therapy and donor-derived bone marrow transplantation results in the repopulation of the host thymus with donor-type dendritic cells (DCs) that delete donor-reactive T cells. Complete chimerism is the replacement of all host hematopoietic cells with donor-derived stem cells such as hematopoietic stem cells (HSCs). Because such donor-derived stem cells have the ability to replicate perpetually, they theoretically continue to provide donor-type DCs indefinitely.

**FIGURE 1:**
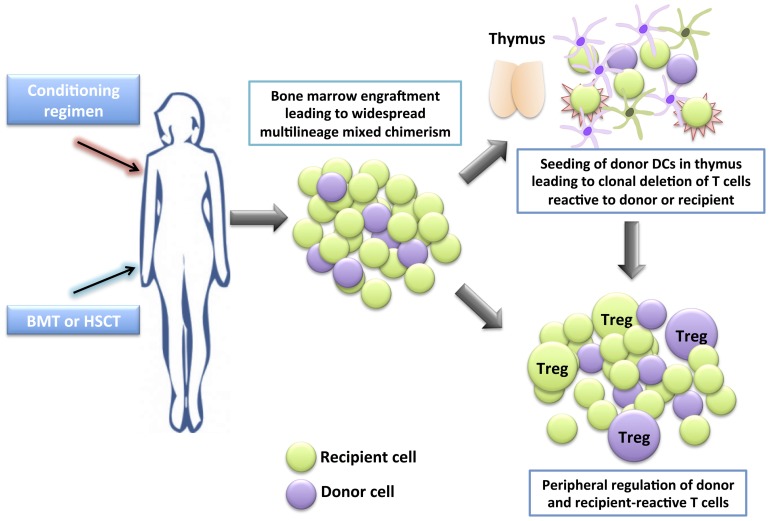
**Development of tolerance through mixed chimerism**. A conditioning regimen is administered which consists of a combination of drugs (occasionally together with irradiation) to allow the engraftment of allogeneic bone marrow (BM) or hematopoietic stem cells (HSCs). A bone marrow transplant (BMT) or hematopoietic stem cell transplant (HSCT) is given the to patient. The donor HSCs seed into the recipient’s BM niches together with the recipient’s HSCs, providing a self-renewing source of donor and recipient hematopoietic cells, leading to widespread multilineage mixed chimerism. Donor dendritic cells seed the thymus, and together with recipient dendritic cells, mediate central clonal deletion of newly developing donor-reactive and recipient-reactive thymocytes. Peripheral regulation also takes place whereby newly developing donor-reactive and recipient-reactive T cells that escape negative selection in the thymus are suppressed in the periphery by regulatory T cells (Treg).

A number of successful clinical cases in SOT have been reported whereby patients with hematological indications for bone marrow ablation who also require renal transplantation have received a BMT and a kidney transplant from the same donor, resulting in long-term donor-specific tolerance (Buhler et al., 2002; Fudaba et al., 2006; Spitzer et al., 2011). Nevertheless, the morbidity and mortality of myeloablative therapy and risk of graft-versus-host disease (GvHD) in most transplant recipients makes this mode of therapy unacceptable to those without a hematological indication for bone marrow ablation. On the other hand, mixed chimerism, where donor cells represent a varying proportion (but not 100%) of the total hematopoietic pool is a more promising area of research (Kawai et al., 2011). Mixed chimerism can be established using non-myeloablative conditioning regimens, therefore maintaining immunocompetence and reducing the risk of GvHD (**Figure 1**).

There is evidence for the operation of both central deletional and peripheral regulatory mechanisms in mixed chimerism (Pilat and Wekerle, 2010; Sachs et al., 2011). In models where mixed chimerism is induced following total body irradiation (TBI), the specific depletion of donor cells is associated with the appearance of donor-reactive T cells in the periphery and the loss of tolerance (Khan et al., 1996). This loss of tolerance may be avoided by removal of the thymus before depletion of donor cells, highlighting the importance of the intrathymic chimerism in the maintenance of tolerance. In less intensive conditioning mechanisms where costimulatory blockade is used to facilitate mixed chimerism, intrathymic deletion remains an important mechanism contributing to tolerance and there is no evidence of a role for Treg (Wekerle et al., 2000; Fehr et al., 2008). Nevertheless, regimens that produce less complete deletion of pre-existing donor-reactive T cells may be dependent on peripheral tolerance mechanisms. For example, in a costimulation-based non-myeloablative BMT model, depletion of CD25^+^ cells at the time of BMT prevents the induction of tolerance (Bigenzahn et al., 2005). CD4^+^ T cells isolated from these chimeras display regulatory capabilities. In another model, the challenge of mixed chimeras with naïve T cells does not lead to the rejection of skin allografts, suggesting a role for peripheral regulatory mechanisms (Domenig et al., 2005). Moreover, mixed chimerism induction techniques that actively employ peripheral regulation, for example, by the infusion of Treg, may facilitate the development of mixed chimerism and lead to more robust tolerance (Seung et al., 2003; Pilat et al., 2010).

A series of promising clinical trials for SOT utilizing mixed chimerism for the induction of tolerance have been performed. An initial trial enrolled six patients with renal failure consequent to multiple myeloma (Fudaba et al., 2006). Patients received non-myeloablative BMTs and renal transplants from an HLA-identical sibling followed by a donor leukocyte infusion as treatment for both the multiple myeloma and renal failure. Four patients transiently developed mixed chimerism, which was later lost, while the other two patients eventually developed full donor chimerism. Interestingly, all patients successfully accepted their renal transplants long-term (up to >9 years) without any immunosuppression. Following this study, a similar approach was piloted in five patients without a hematological malignancy (Kawai et al., 2008; LoCascio et al., 2010). Patients received an HLA-mismatched haploidentical bone marrow transplant along with a renal transplant from the same donor. All patients developed transient mixed chimerism, but this was lost after day 21. Four patients in the trial currently maintain graft function after weaning from their initial immunosuppression (follow-up 2–5 years post-weaning). However, one kidney graft was lost due to acute antibody-mediated rejection, leading to a modification in the trial protocol to include B cell depletion with rituximab.

In another recent proof-of-concept study, 12 living donor HLA-matched kidney transplant recipients received a donor-cell infusion of 5–16 × 10^6^/kg CD34^+^ cells mixed with 1–10 × 10^6^/kg CD3^+^ T cells after conditioning with total lymphoid irradiation and five doses of rabbit antithymocyte globulin (Scandling et al., 2011), as per a protocol previously reported as a case report (Scandling et al., 2008). None of the patients developed GvHD. In 8 of the 12 patients, maintenance immunosuppression was eventually discontinued and patients have since been immunosuppression-free with good graft function and without evidence of acute or chronic rejection (follow-up 12–36 months). The remaining four patients experienced rejection episodes on weaning from immunosuppression and have therefore remained on immunosuppression. The authors now plan to apply the protocol to HLA-mismatched transplant recipients. Given the notable immunosuppression-free success rate and safety profile, it will be interesting to observe the efficacy of the protocol in this situation.

A recent Phase II clinical trial investigated the use of HSCT together with a facilitating cell (FC) infusion to promote the development of chimerism and subsequent tolerance in eight HLA-mismatched living donor renal transplant recipients (Leventhal et al., 2012). FCs were defined as CD8^+^ bone marrow-derived cells that did not express the TCR and primarily contained a plasmacytoid DC population (Kaufman et al., 1994; Grimes et al., 2004; Fugier-Vivier et al., 2005). In this clinical trial, combined FC and HSCT transplantation initially led to high levels of chimerism in all eight renal allograft recipients. Five of the recipients displayed stable chimerism and donor-specific tolerance and were subsequently weaned off maintenance immunosuppression one year post-transplant. Two patients developed only transient chimerism and were therefore maintained on low-dose tacrolimus monotherapy. One patient, although displaying robust chimerism, developed viral sepsis 2 months post-transplantation and subsequently lost the kidney graft due to renal artery thrombosis. Immunological monitoring of the patients enrolled in the trial showed a significant reduction in circulating CD4^+^ but not CD8^+^ cells post-transplantation. The significance of this observation is unclear, however it may be indicative of central deletion of alloreactive CD4^+^ T cells or peripheral regulation by Treg. Indeed, an increase in the Treg to effector T cell ratio was observed in chimeric recipients but not those that achieved only transient macrochimerism.

While the above approaches have demonstrated some success in living donor transplants, the induction of mixed chimerism in recipients of cadaveric organ transplants may prove more challenging. BMT has only been trialed on a small scale in cadaveric donor transplants, principally in the context of vascularized composite allograft (VCA) transplants. The first face transplant performed in France used a post-transplant donor-derived bone marrow infusion, although it does not appear that this approach accorded any clear benefit in terms of a reduction in episodes of rejection (Hequet et al., 2008). Furthermore, microchimerism was only detectable at a single point 2 months post-operatively and not thereafter (Hequet et al., 2008). Since then, five VCA transplants performed in Pittsburgh have employed the “Pittsburgh Protocol” in which a bone marrow infusion is given within 15 days of VCA transplantation (*International Hand and Composite Tissue Allotransplantation Society Congress, Atlanta 2011)*. Early reports indicate that patients treated in this manner have been maintained successfully on single drug immunosuppression with tacrolimus.

Interestingly, the presence of vascularized bone marrow in many VCA transplants raises the possibility that chimerism may develop by nature of the simultaneous transplantation of HSCs within the VCA. To this end, various rat models of hindlimb transplantation using T cell depleting antibody along with immunosuppression have achieved long-term allograft survival (Siemionow et al., 2002a,b, 2003; Ozer et al., 2003, 2004; Siemionow and Klimczak, 2009), although mixed chimerism is not always readily detectable (Quatra et al., 2006). In these models, the bone marrow component of the VCA transplant is critical to the attainment of mixed chimerism and long-term allograft survival (Siemionow et al., 2005; Siemionow, 2011). Moreover, increased levels of chimerism are detectable with larger sized VCA allografts in rats, indicating the role of the transplant in providing donor cells (Nasir et al., 2008). In a rat model of facial allograft transplantation, mixed macrochimerism has been observed with the use of only cyclosporine monotherapy (Demir et al., 2004; Kulahci et al., 2010). Mixed chimerism has also been achieved using non-depleting CD4^+^ blockade and depleting CD8^+^ antibody in conjunction with rapamycin and α-CD154 costimulatory blockade, without a bone marrow transplant, relying on bone marrow in a mouse hindlimb VCA to provide donor cells for chimerism (Li et al., 2008). In general, clinical data have not been particularly encouraging, with no evidence for the development of chimerism in VCA transplantation. This may be due to only small amounts of bone marrow being transferred that have limited functionality in the adult (Granger et al., 2002; Petruzzo et al., 2003). Moreover, there is experimental evidence that the recipient thymus is necessary for peripheral chimerism to develop after transplantation of a bone marrow-containing VCA (Li et al., 2007). In humans the thymus involutes and becomes atrophic after puberty and is therefore less likely to support the development of chimerism. It is important to note that in theory, chimerism is a double-edged sword, whereby the greater the likelihood of chimerism, the greater the anti-host alloresponse and risk of GvHD (Wood, 2003).

## REGULATORY T CELLS

While the methods described above relate to exploiting the natural mechanisms used by the immune system to ensure self-tolerance through central mechanisms, several peripheral regulatory mechanisms also exist as a fail safe mechanism to maintain self-tolerance and to prevent an overshoot of the normal immune response (Issa and Wood, 2010; Wood and Goto, 2012). While most autoreactive cells are deleted centrally in the thymus, some autoreactive T cells escape this process and require peripheral regulation to prevent autoimmunity. CD4^+^ Treg are central to these mechanisms. *Scurfy* mice lacking the Treg-specific transcription factor forkhead box P3 (foxp3) develop a lymphoproliferative disorder (Brunkow et al., 2001) and humans with mutations in *FOXP3* can develop IPEX (immunodysregulation, polyendocrinopathy, and enteropathy, X-linked; Bennett et al., 2001). *FOXP3* is closely linked to suppressive activity and its sustained expression is required for the maintenance of regulatory activity (Josefowicz and Rudensky, 2009).

Treg can be divided into thymus-derived naturally occurring CD4^+^CD25^hi^FOXP3^+^ Treg (nTreg or tTreg; Hori et al., 2003) and induced or adaptive CD4^+^ Treg (iTreg), which upregulate FOXP3 in the periphery under defined conditions of antigen-exposure, for example, in the presence of transforming growth factor β (TGFβ; Kingsley et al., 2002; Karim et al., 2004, 2005). Type 1 Treg (Tr1) cells are a distinct population of peripherally induced Treg that develop in the presence of IL-10 and regulate responses through FOXP3-independent secretion of IL-10 and TGFβ, leading to bystander regulation of effector T cells (Battaglia et al., 2006a). nTreg represent 5–10% of the peripheral CD4^+^ pool and constitutively express high levels of surface CD25 although this is not a reliable marker due to its upregulation on recently activated T cells. Nevertheless, although CD25 appears on recently activated CD4^+^ T cells, some of these are true proliferating Treg. For example, during the secondary antigenic response that develops after human tuberculin purified protein derivative is injected into skin, CD4^+^CD25^+^ cells proliferate within the skin. Many of these proliferating cells are in fact FOXP3^+^ and display functional and phenotypic markers of Treg (Vukmanovic-Stejic et al., 2008). It is unclear how much of the peripheral CD4^+^ population iTreg represent, but given that these cells are induced in specific inflammatory environments it is likely that their number is location and time-dependent.

In transplantation, both direct and indirect allorecognition contribute to the immune response that results in graft destruction. However, with time after transplantation, passenger antigen-presenting cells are lost and organ parenchyma is less able to stimulate the host via the direct pathway. The indirect alloresponse therefore becomes of increasing importance and may be more relevant in chronic rejection (Baker et al., 2001). Interestingly, alloreactive T cells that respond by the indirect pathway are more resistant to inhibition by conventional immunosuppression and are detectable in the peripheral blood of transplant recipients years after transplantation (Sawyer et al., 1993; Vella et al., 1997). The alloreactivity of Treg may therefore be important in determining their ability to promote tolerance. Indeed, Treg that are both directly and indirectly alloreactive are able to prevent both acute and chronic rejection in mice, whereas those that are only directly alloreactive appear to only be able to prevent acute rejection (Joffre et al., 2008; Tsang et al., 2008).

Studies assessing the potential of nTreg, iTreg, and Tr1 cells to promote allograft survival in experimental transplantation have yielded promising results to date. In these studies Treg may be induced *in vivo* by employing costimulatory blockade or lymphocyte depletion around the time of transplantation, often together with an antigen challenge (Cobbold et al., 1986; Qin et al., 1993; Graca et al., 2000; Kingsley et al., 2007; Francis et al., 2011). Alternatively nTreg may be expanded *ex vivo* or converted from non-Treg cell types to iTreg *in vitro*.

## *EX VIVO* EXPANSION

Human Treg for cell therapy protocols are produced by isolation of cells from peripheral or umbilical cord blood (UCB) and subsequent *ex vivo* expansion or direct use *in vivo*. In order to isolate Treg efficiently and to a high purity, reliable markers of identification are required. Given the non-exclusivity of CD25 and FOXP3 expression, a number of other markers are in use. Of these, CD127 (the IL-7 receptor α-chain), CD49b (the α-chain of the integrin VLA-4 – a4b1), CD45RA, and latency-associated peptide (LAP) are particularly useful. Other Treg markers include CD152 (CTLA-4), GITR, CD69, and CD44 but these are less useful as they may also be expressed in almost identical patterns on non-regulatory activated T cells.

The use of the low expression of CD127 for the isolation of Treg was described approximately 5 years ago (Liu et al., 2006; Seddiki et al., 2006; Putnam et al., 2009) and is particularly helpful as it defines a highly suppressive population of Treg. In a humanized mouse model of vessel allograft rejection, human *ex vivo-expanded* CD25^hi^CD4^+^ or CD127^lo^CD25^+^CD4^+^ nTreg were used to modulate immune responses *in vivo* to reduce neointimal expansion. Treg expressing low levels of CD127 were found to be five times more potent than those expressing only CD25. The same population of CD127^lo^Treg have been shown to be active in the prevention of human skin graft rejection in a similar humanized mouse model (Issa et al., 2010). The absence of CD49b is another helpful marker for Treg identification, as together with CD127 it allows for Treg isolation by negative selection alone (Kleinewietfeld et al., 2009).

CD45RA allows cells to be divided into CD25^+^CD45RA^+^FOXP3^lo^ (resting naïve Treg), CD25^hi^CD45RA^–^FOXP3^hi^ (activated Treg), and CD25^+^CD45RA^–^FOXP3^lo^ (non-suppressive T cells) populations (Miyara et al., 2009). Resting naïve and activated Treg are both suppressive *in vitro*, whilst only resting naïve Treg proliferate *in vivo* and evolve into suppressive CD45RA^–^Treg. UCB contains a high number of naïve CD45RA^+^ cells, and is therefore an attractive source of resting naïve Treg (Riley et al., 2009). However, UCB Treg are low in frequency and require either *in vitro* culture or pooling of multiple blood units. Furthermore, as UCB Treg are allogeneic to both the donor and recipient they are likely to be subject to an alloresponse therefore complicating their *in vivo *use.

LAP has been shown to define a population of Treg that express high levels of foxp3, secrete immunosuppressive TGFβ, and exhibit enhanced *in vivo* regulatory activity (Chen et al., 2008). Moreover, LAP itself is functionally suppressive independent of TGFβ (Ali et al., 2008). In Treg expansion cultures, the expression of LAP allows the distinction and selection of activated Treg from activated non-Treg cell types (Tran et al., 2009).

*Ex vivo* expansion of isolated Treg is largely performed by stimulation with αCD3/αCD28 microbeads in the presence of recombinant human (rh) IL-2 (Sagoo et al., 2008; Trzonkowski et al., 2009). The non-specific TCR stimulation in this system leads to the production of a polyclonally reactive population of Treg. Donor alloantigen-reactive Treg that have been expanded in the presence of donor-derived APC have been shown to be more potent suppressors *in vitro *and *in vivo *than polyclonally reactive Treg, and their specific reactivity implies that they are safer for *in vivo* use (Golshayan et al., 2007; Sagoo et al., 2011). Selection of alloantigen-stimulated Treg from a culture where allogeneic stimulators are used may be possible by enrichment of Treg that co-express the activation markers CD69 and CD71 (Sagoo et al., 2011; J. Hester and K.J. Wood, unpublished data). Other methods for the production of alloantigen-reactive Treg include retroviral vector transduction of Treg with genes that encode for TCRs with known antigen specificities (Jiang et al., 2006).

### *IN VIVO* INDUCTION

*In vivo* approaches are based on increasing the frequency or potency of Treg by exposure to antigen, inducing an expansion of nTreg or converting non-Treg to iTreg (Long and Wood, 2009; Francis et al., 2011). Treg may be generated *in vivo* by pre-treating mice with a donor alloantigen (in the form of a donor-specific transfusion) along with a non-depleting α-CD4 mAb (Kingsley et al., 2002, 2007). Treg produced in this manner are capable of preventing allograft rejection *in vivo *(Wood et al., 1991; Bushell et al., 1994, 1995; Saitovitch et al., 1995, 1996, 1997; Kingsley et al., 2002, 2007; Bushell et al., 2003; Karim et al., 2005; Warnecke et al., 2007). While this tolerance appears to be antigen-specific in nature, another “boosting” blood transfusion allows tolerance to develop to a third-party allograft (Karim et al., 2005). CD4^+^CD25^+^ Treg isolated from these animals may prevent allograft rejection in naïve mice by adoptive transfer (Hara et al., 2001; Kingsley et al., 2002) and are able to prevent skin graft rejection initiated by both CD4^+^ (Hara et al., 2001; Golshayan et al., 2007) and CD8^+^ (van Maurik et al., 2002; Jones et al., 2010) T cells. Alternatively, costimulatory blockade or lymphocyte depletion using monoclonal antibodies around the time of transplantation may also promote tolerance induction (Qin et al., 1990; Qin et al., 1993; Graca et al., 2000; Waldmann et al., 2006). Interestingly, even nTreg isolated from naïve animals may prevent rejection, although 10-fold more such Treg are required to attain long-term allograft survival compared to Treg isolated from tolerant mice treated with antigen exposure (Graca et al., 2002). The folate receptor 4 (FR4) allows the identification of these alloantigen-stimulated Treg (Yamaguchi et al., 2007). Alloantigen-stimulated FR4^high^ Treg are significantly more effective at prolonging mouse skin allograft survival compared to FR4^intermediate^ Treg.

Another method for *in vivo* generation is the injection of IL-2-IL-2 mAb complexes into mice, resulting in an over 10-fold expansion of Treg *in vivo*. Animals treated by this method are resistant to experimental autoimmune encephalomyelitis (EAE) induction and display tolerance to islet allografts (Webster et al., 2009). Injection of IL-2-IL-2 mAb complexes together with recombinant granulocyte-colony stimulating factor (G-CSF) induces expansion of Treg and myeloid-derived suppressor cells (MDSCs) *in vivo*, promoting mouse skin allograft survival in MHC Class II-mismatched models (Adeegbe et al., 2010).

There has been a great deal of discussion regarding the functional stability of Treg. Zhou et al. (2009) demonstrated that some Treg lose foxp3 (becoming exfoxp3 cells), developing an activated memory-type phenotype, and are pathogenic *in vivo*. This loss of foxp3 was linked to a proinflammatory microenvironment in which Treg acquire an effector T cell phenotype, secreting IL-17 and interferon γ (IFNγ; Yang et al., 2008; Ayyoub et al., 2009; Komatsu et al., 2009; Voo et al., 2009; Chadha et al., 2011). Importantly, Treg may not be particularly effective at suppressing IL-17 producing T cells (Heidt et al., 2010; Zhang et al., 2010; Chadha et al., 2011), although data from our own studies in kidney transplant patients treated with the leukocyte depleting monoclonal antibody alemtuzumab (Campath or anti-CD52 antibody) suggest that Treg present in these patients can regulate Th17 cells (Hester et al., 2011). Stability of Treg is important to consider in transplantation as there is evidence that Treg transfer into lymphopaenic mice may result in the loss of foxp3 expression in up to 50% of the adoptively transferred cells (Duarte et al., 2009). This is a particularly important point to consider if Treg cellular therapy is to be employed in patients who are lymphopaenic post-immunosuppressive induction therapy. However, a recent study by Miyao et al. (2012) has elegantly demonstrated that the plasticity of Treg is due to only a minor population of foxp3^+^ cells. In this study, suppressive foxp3^+^Treg do not develop effector cell function even in inflammatory or lymphopaenic environments. While such Treg may transiently lose foxp3 expression, on activation foxp3 is re-expressed and suppressive capabilities return. It is therefore only a small minority (2–3%) of the peripheral foxp3^+^ pool which are originally non-regulatory and which may then lose foxp3 to become pathogenic. Overgrowth of this small population may explain previous data demonstrating the plasticity of Treg. Importantly in this study, it is epigenetic control of Foxp3 that dictates whether foxp3^+^ cells are true Treg. Demethylation of the Treg cell-specific demethylated region (TSDR) indicates that cells are committed suppressive Treg, regardless of the ongoing expression of foxp3. Identification of the methylation status of the TSDR is therefore a valuable indicator of the purity of cell preparations produced for clinical use.

## REGULATORY B CELLS

There are multiple reports of clinical operational tolerance, or long-term functioning allograft survival in the absence of any immunosuppression. This has most commonly been observed in liver transplantation (Lerut and Sanchez-Fueyo, 2006) but has also been reported in a small number of renal transplant recipients (Orlando et al., 2010). In a study where the immune profile of tolerant renal transplant recipients was analyzed, the most striking feature was a bias towards a differential expression of B cell-related genes and an expansion of peripheral blood B cells in tolerant patients (Newell et al., 2010; Sagoo et al., 2010). This latter observation raises the interesting possibility that regulatory B cells (Bregs) may be playing a role. Bregs express high levels of CD1d, CD21, CD24, and IgM, have an immature or transitional phenotype, and are active through the secretion of suppressive IL-10 (Mauri and Blair, 2010). IL-10-secreting B cells have been shown to regulate autoimmune responses *in vivo *(Fillatreau et al., 2002; Mauri et al., 2003). There are currently no clinical studies examining Bregs as a cellular therapy. Further work is required to determine the optimal methods for the production of a functionally suppressive population of Bregs that may be used clinically.

## DENDRITIC CELLS

While DCs are known to be pivotal in the development of the alloresponse, some populations of DCs may also be active in the promotion of tolerance (Morelli and Thomson, 2007; van Kooten et al., 2011). Immature myeloid-derived DCs have been shown to promote the survival of heart allografts in a donor-specific manner (Lutz et al., 2000) and regulatory DCs with low costimulatory ability may prevent the development of GvHD in mice (Sato et al., 2003). However, even mature DCs expressing normal or high levels of MHC and costimulatory molecules may promote the development of tolerance. These DCs prime CD4^+^ and CD8^+^ T cells that in turn develop regulatory activity *in vitro *(Albert et al., 2001; Verhasselt et al., 2004). *In vivo*, myeloid-derived DCs matured with tumor necrosis factor α (TNFα) and expressing high levels of MHC II are able to protect mice from CD4^+^ T cell-mediated EAE (Menges et al., 2002).

The tolerogenic effects of DCs can be potentiated by the administration of costimulatory blockade agents. For example, plasmacytoid DCs have been shown to promote the induction of IL-10-secreting Treg and may promote heart allograft survival *in vivo *(Gilliet and Liu, 2002; Abe et al., 2005). However, with the addition of anti-CD154 antibody, this effect is significantly enhanced (Bjorck et al., 2005). A similar effect may be observed with the administration of costimulatory blockade with immature myeloid-derived DCs (Lu et al., 1997). Plasmacytoid DCs may also be important in facilitating mixed chimerism as described earlier when in the form of FCs (Kaufman et al., 1994; Grimes et al., 2004; Fugier-Vivier et al., 2005; Leventhal et al., 2012). Importantly, FCs have been demonstrated to promote the generation of Treg and prevent GvHD development in mice (Colson et al., 2004; Taylor et al., 2007; Huang et al., 2011).

## MYELOID-DERIVED SUPPRESSOR CELLS AND REGULATORY MACROPHAGES

Myeloid-derived suppressor cells are a heterogeneous population of cells with both innate and adaptive immune targets, which include T, B and NK cells (Boros et al., 2010). Common phenotypic markers among MDSCs include GR1 and CD11b in mice and CD33, CD11b, CD34, and low MHC Class II expression in humans. Experimentally, mouse MDSCs induced by costimulatory blockade *in vivo* migrate to heart transplants where they prevent the development of alloresponses and promote the development of Treg (Garcia et al., 2010). Similarly, anti-CD28 antibody-induced rat kidney allograft tolerance leads to the accumulation of MDSCs in the blood. These MDSCs inhibit effector T cell proliferation *in vitro *through the activity of inducible nitric oxide (NO) synthase. Interestingly, however, the adoptive transfer of MDSC in this model does not induce kidney allograft tolerance.

Regulatory macrophages (Mregs) are a population of macrophages which produce large amounts of IL-10 and are able to suppress T cell proliferation *in vitro *(Fleming and Mosser, 2011). A population of cells termed “transplant acceptance inducing cells” (TAICs) has also been shown to promote the survival of heart and lung transplants in animal models (Fandrich et al., 2002a,b,c). TAICs are impure populations of macrophages contaminated with other leukocytes, whereas Mreg preparations are a uniform population of macrophages (Hutchinson et al., 2012).

## CLINICAL TRIALS OF CELLULAR THERAPY FOR PERIPHERAL REGULATION

Several studies have investigated the use of Treg for the treatment of GvHD post-HSC transplantation. These studies are paving the way for Treg therapy in SOT. Trzonkowski et al. (2009) reported the “first-in-man” trial of *ex vivo*-expanded recipient-derived Treg in two patients: in one case of chronic GvHD a significant alleviation of symptoms and a reduction of required immunosuppression was achieved, whereas in one case of severe grade IV acute GvHD only a transient improvement in symptoms and signs was reported.

Significantly, two major Phase I/II trials have been carried out at the University of Minnesota and in Italy. Blazar’s group in Minnesota evaluated the safety profile of human UCB-derived partially HLA-matched *ex vivo*-expanded Treg (Brunstein et al., 2011). The study was designed as a Phase I dose-escalation trial and reported a reduced incidence of grades II–IV acute GvHD in the test group of 23 patients compared to 108 identically treated historical controls not receiving Treg therapy. Doses of Treg ranged from 1 × 10^5^/kg to 30 × 10^5^/kg and there was no reported increase in infectious complications. The Italian study was performed to assess the safety and efficacy of expanded CD4^+^CD25^+^ human nTreg in prevention of GvHD in 28 patients with high-risk acute leukaemias undergoing HLA-haploidentical HSC transplants. Patients were also given donor conventional T cells to enhance immune reconstitution. Treg were derived from the same HLA-haploidentical donor by apheresis followed by large scale CD4^+^CD25^+^ magnetic bead selection. Despite no GvHD prophylaxis being given, chronic GvHD did not develop in 26 out of 28 patients in whom full donor-type engraftment was achieved. However, in two of the 26 patients acute GvHD of grade II or above developed, which may be due to these two patients being given the highest dose of conventional T cells. Patients in the Italian trial displayed an overall faster post-transplant immune reconstitution as well as a reduction in the risk of CMV reactivation compared to those not receiving Treg (Di Ianni et al., 2011). While Brunstein et al. (2011) used UCB-derived Treg, Di Ianni et al. (2011) used adult expanded Treg. The difference in efficacy between these two populations on a cell-by-cell basis is unclear from these studies. However, as discussed earlier, UCB-derived Treg may contain a higher proportion of “naïve” CD45RA^+^ Treg and therefore a greater number of Treg which may readily proliferate *in vivo*. This would theoretically represent an advantage in terms of the cell dose required to prevent disease.

Other ongoing trials not yet published include one being conducted by Matthias Edinger at the University Hospital in Regensburg using CD25^hi^ magnetically isolated non-expanded Treg infused into post-HSCT recipients (Edinger and Hoffmann, 2011). Trials using Tr1 cells for GvHD are also ongoing at the San Raffaele Hospital in Milan. Early results have been promising with no adverse side effects (Roncarolo and Battaglia, 2007; Allan et al., 2008; Battaglia and Roncarolo, 2011).

Two Phase I/II trials of TAICs assessed the safety of administration of these cells in 5–12 kidney transplant patients (Hutchinson et al., 2008a,b, 2009). The studies aimed to determine the possibility of immunosuppression withdrawal. The infusion of TAICs appeared safe but did not promote tolerance, with acute rejection developing in several patients on withdrawal of immunosuppression. Nevertheless, renal function was maintained in four out of five patients that were tapered to low-dose tacrolimus monotherapy. Moreover, one patient achieved complete immunosuppression withdrawal for 8 months before experiencing a rejection episode. Moving on from this approach, more uniform Mreg populations have been trialed in two renal transplant recipients, leading to a reduction in the required dose of immunosuppression with good graft function at 3 years post-transplantation (Hutchinson et al., 2011).

The European Union is currently funding the first study for the evaluation of immunomodulatory cellular therapy in SOT (www.onestudy.org). The *ONE Study*, a multicenter Phase I/II clinical trial, will evaluate the safety and feasibility of various types of cell therapy including expanded nTreg, Tr1 cells, Mregs, and tolerogenic DCs in living-donor kidney transplantation. All centers will utilize a common adjunctive immunosuppressive protocol in order to provide a true comparison of the various cellular therapies. Control patients will be transplanted in 2013 and cell therapy groups in 2014, providing a follow-up period of 12 months. **Table 1** summarizes the concluded and ongoing clinical trials of cellular therapy.

**Table 1:** Concluded and ongoing clinical studies using cellular therapy for peripheral regulation.

Group	Number of patients	Condition	Therapy	Outcome
Trzonkowski (Trzonkowski et al., 2009)	2	HLA-matched BMT or HSCT	Expanded CD4^+^CD25^+^CD127^–^ donor Treg as treatment for GvHD	Patient 1: Reduction of immunosuppression Patient 2: Transient clinical improvement
Martelli (Di Ianni et al., 2011)	28	HLA-haploidentical HSCT	Freshly isolated CD4^+^CD25^+^ donor Treg	Low incidence of acute and chronic GvHD with improved immune reconstitution
Blazar (Brunstein et al., 2011)	23	Double unit unrelated UCB	Expanded CD4^+^CD25^+^ third-party UCB Treg as prophylaxis against GvHD	Reduced incidence of grade II-IV GvHD
Trzonkowski (Trzonkowski et al., 2011)	4	BMT/HSCT	Expanded CD4^+^CD25^+^CD127^–^ donor Treg as treatment for GvHD	Alleviation of one case of chronic GvHD, no effect on acute GvHD
Edinger (Edinger and Hoffmann, 2011)	9	HSCT	Freshly isolated Treg	Ongoing: appears safe and feasible
Roncarolo (Battaglia and Roncarolo, 2011)	16	HLA-haploidentical HSCT	Allostimulated donor Tr1 cells	Ongoing: appears safe and feasible
Geissler/Fandrich (Hutchinson et al., 2009)	1	Deceased donor kidney transplant	TAICs: feasibility study	Immunosuppression reduced to low-dose tacrolimus therapy, safe and feasible
Geissler/Fandrich (Hutchinson et al., 2008b)	12	Deceased donor kidney transplants	TAICs	No clear benefit, but safe and feasible
Geissler/Fandrich (Hutchinson et al., 2008a)	5	Living-donor kidney transplants	TAICs	Four patients tapered to low-dose tacrolimus monotherapy but higher rate of early acute rejection
Geissler/Fandrich (Hutchinson et al., 2011)	2	Living-donor kidney transplants	Mregs	Both patients tapered to low-dose tacrolimus monotherapy
The One Study (Wood et al., 2012)	Recruiting	Living-donor kidney transplants	Treg, Mreg, Tr1 cells, tolerogenic DCs	Ongoing feasibility study
Bluestone/Herold (Gitelman et al., 2012)	Recruiting (14 patients)	Treatment of type 1 diabetes	Autologous expanded CD4^+^CD25^+^CD127^–^Treg	Ongoing feasibility study

## IMMUNOTHERAPY FOR THE PROMOTION OF TOLERANCE

Treg and effector T cells preferentially employ different intracellular activation pathways. Treg utilize IL-2-dependent STAT-5 (Burchill et al., 2007; Vogtenhuber et al., 2010), whereas effector T cells utilize the phosphoinositide 3-kinase/Akt/mTOR pathway (Delgoffe et al., 2009). Rapamycin, an mTOR inhibitor, takes advantage of this distinction. The beneficial effects of rapamycin on Treg survival and proliferation have been demonstrated *in vitro* and *in vivo *(Battaglia et al., 2005, 2006b,c; Gao et al., 2007; Zeiser et al., 2008; Hendrikx et al., 2009). Experimental work in mouse allograft models has demonstrated that rapamycin inhibits chronic cardiac allograft rejection and that this effect is potentiated when used in combination with an α-CCR5 antibody (Li et al., 2009). In this study, an increase in intragraft numbers of CD4^+^CD25^+^foxp3^+^ Treg was observed. A similar effect has also been observed clinically, with rapamycin increasing the frequency of CD62L^high^ Treg in the peripheral blood of lung transplant recipients (Lange et al., 2010). Experimentally, the adoptive transfer of a small number of alloantigen-specific Treg along with low dose rapamycin treatment has been shown to induce long-term survival of cardiac allografts in mice (Raimondi et al., 2010). Moreover, alloantigen-pulsed rapamycin-conditioned DCs have been shown to promote long-term engraftment of vascularized skin allografts in rats with an associated expansion of CD4^+^foxp3^+^ Treg (Horibe et al., 2008).

Rabbit anti-murine thymocyte globulin (mATG), a T cell depleting polyclonal antibody has also shown promise. ATG promotes the generation of Treg (Lopez et al., 2006), and when combined with CTLA4-Ig and rapamycin, mATG shifts the effector memory T cell-Treg balance in favor of Treg, prolonging the survival of skin allografts in a fully MHC-mismatched mouse model (D’Addio et al., 2010).

Interestingly, glucocorticoids may act on human Langerhans cells to promote a phenotype that favors the induction of Treg *in vitro *(Stary et al., 2011). Some patients treated with glucocorticoids have increased numbers of dermal FOXP3^+^CD25^+^ Treg as well as increased numbers of epidermal Langerhans cells that display upregulated expression of TGFβ mRNA. However, there is no clear clinical evidence that ATG or glucocorticoids are beneficial in terms of increasing Treg numbers in transplant recipients.

Alemtuzumab may favor Treg survival, with evidence from one study demonstrating a higher proportional depletion of T effector cells than Treg (Bloom et al., 2008). However, data in this study are confounded by the introduction of rapamycin in patients early after transplantation. Indeed, in a separate study Treg numbers in alemtuzumab-treated patients remained low until the late introduction of rapamycin (Trzonkowski et al., 2008). Interestingly, Bregs have been identified in renal transplant recipients treated with alemtuzumab (Heidt et al., 2012). Alemtuzumab induction has been trialed at the University of Wisconsin for the minimization of immunosuppression (Knechtle et al., 2009). In this study, induction with alemtuzumab together with rapamycin maintenance monotherapy successfully led to long-term graft survival in nine of 10 patients although five patients developed anti-donor antibodies and graft C4d deposition.

Blockade of the IL-2-CD25 or CD28-CD80/CD86 pathways is an effective method of producing T cell anergy experimentally (Vincenti, 2008), however these pathways are also essential for the survival of Treg. Indeed, in mouse models where these pathways are targeted, there is a reduction in the survival and function of Treg with an associated exacerbation of autoimmunity *in vivo *(Tang et al., 2003; Wing et al., 2008). Clinically, however, there is no difference in circulating Treg numbers between renal transplant recipients treated with both belatacept (a second-generation CTLA-4-related. Ig fusion protein) and basiliximab (an α-CD25 monoclonal antibody, mAb) compared to those treated with calcineurin inhibitors (CNIs; Bluestone et al., 2008). Nevertheless, CNIs such as cyclosporine have a detrimental effect on Treg (Ma et al., 2009; Presser et al., 2009), thus confounding this observation. In this same study patients receiving belatacept displayed higher levels of intragraft FOXP3^+^ T cells during acute rejection (Bluestone et al., 2008). Belatacept has proven to be an effective immunosuppressant, but has not yet demonstrated any efficacy in the promotion of transplant tolerance in clinical transplantation. This may be related to the blockade of CD80/86-CTLA-4 interaction by CTLA-4.Ig. In a study investigating a novel CD28 antagonist for use in transplantation, there was an increase in the number and activity of Treg in a non-human primate (NHP) renal transplantation model (Poirier et al., 2010). The benefit of this costimulatory blockade, unlike CTLA-4.Ig, is that it allows physiological immune regulation through CD80/86 to continue (Wing et al., 2008). The effects α-CD25 mAb on Treg are not entirely clear. In the study by Bluestone et al. (2008), basiliximab was shown to deplete all CD25-bearing cells, including Treg. However, in another study examining daclizumab (a humanized α-CD25 mAb) in cardiac transplant patients, Treg generation in the periphery was not affected (Vlad et al., 2007). The timing of treatment with α-CD25 antibodies or CTLA-4.Ig may be of critical importance and may explain some of the differences between data from animal and human studies. Early use of these molecules may target Treg, resulting in deleterious effects in models dependent on Treg function, whereas later use post-transplantation may preferentially target activated effector T cells. Other possibilities include a lower sensitivity of human Treg to CD28 blockade or the presence of other costimulatory molecules on human Treg that may substitute for the absence of CD28 costimulation.

While CNIs are normally detrimental to Treg, there is some evidence that low-dose cyclosporine may enhance the number of Treg in the skin of patients with atopic dermatitis (Brandt et al., 2009). This appears to be related to the retained ability of patients on low-dose cyclosporine to produce IL-2, which is necessary for Treg survival and expansion (Baumgrass et al., 2010; Brandt et al., 2010).

Memory T cells present a formidable barrier to the induction of tolerance in higher mammals (Brook et al., 2006; Ford and Larsen, 2011). A solution to overcoming this barrier is the use of immunosuppressants that target memory T cell responses while promoting the generation of immunoregulatory elements. In this respect, targeting adhesion molecules such as CD2 or LFA-1 is a promising strategy. Alefacept, an LFA-3.Ig fusion protein binds to and polymerizes CD2, leading to selective elimination of memory T cells. Treatment together with CTLA-4.Ig prevents acute rejection and allows prolonged engraftment of kidney transplants in a NHP model (Weaver et al., 2009). Efalizumab, an anti-LFA-1 antibody, initially displayed promise in early clinical trials of islet transplantation (Badell et al., 2010; Posselt et al., 2010; Setoguchi et al., 2011). Its use however is no longer possible due to withdrawal from the market after the development of progressive multifocal leukoencephalopathy in four patients treated for psoriasis with efalizumab (Tavazzi et al., 2011).

## CONCLUSION

With the trailblazing work of Medawar, clinical tolerance appeared to be eminently within reach. Yet 70 years on, tolerance has been achieved in only a small number of patients in whom full or mixed chimerism was generated. We propose that achieving tolerance in each and every transplant recipient will require a more complete understanding of the biovariability between patients that allows tolerance to be easily induced in some but not others. The attainment of tolerance in a heterogeneous population of transplant recipients may therefore require a tailored approach, with the balanced use of both central and peripheral tolerance induction techniques.

## Conflict of Interest Statement

The authors declare that the research was conducted in the absence of any commercial or financial relationships that could be construed as a potential conflict of interest.
